# Euthanasia

**Published:** 1881-02

**Authors:** Geo. G. Gale

**Affiliations:** Quebec, Canada


### EUTHANASIA.

BY GEO. G. GALE, C.M., M.D., QUEBEC, CANADA.

MALE, about thirty years of age, last stage of consumption; troubled with diarrhœa and *severe* colic. The allopathic doctor has been prescribing astringents and opium. This intoxicates him; he wakes up with his pains increased; his head aches and his mind is dazed from the effects of the opium.

*Colocynth* 200, in water removed the colic, but produced no headache, etc. The man died *without* pain three weeks after. In another case similar to the above, *colocynth* removed the pains and the patient died three days after without experiencing a return of the pains.

